# BRCAness digitalMLPA profiling predicts benefit of intensified platinum-based chemotherapy in triple-negative and luminal-type breast cancer

**DOI:** 10.1186/s13058-020-01313-7

**Published:** 2020-07-25

**Authors:** Esther H. Lips, Anne Benard-Slagter, Mark Opdam, Caroline E. Scheerman, Jelle Wesseling, Frans B. L. Hogervorst, Sabine C. Linn, Suvi Savola, Petra M. Nederlof

**Affiliations:** 1 Department of Molecular Pathology, The Netherlands Cancer Institute, Plesmanlaan 121, 1066 CX Amsterdam, The Netherlands; 2 Department of Oncogenetics, MRC Holland, Amsterdam, The Netherlands; 3 Department of Pathology, The Netherlands Cancer Institute, Amsterdam, The Netherlands; 4 Department of Pathology, Leiden University Medical Center, Leiden, The Netherlands; 5 Department of Medical Oncology, The Netherlands Cancer Institute, Amsterdam, The Netherlands; 6 Department of Pathology, University Medical Center Utrecht, Utrecht, The Netherlands

**Keywords:** Breast cancer, *BRCA1*, *BRCA2*, Copy number analysis, digitalMLPA, Chemotherapy prediction, Genetic analysis, Predictive biomarker

## Abstract

**Background:**

We previously showed that BRCA-like profiles can be used to preselect individuals with the highest risk of carrying *BRCA* mutations but could also indicate which patients would benefit from double-strand break inducing chemotherapy. A simple, robust, and reliable assay for clinical use that utilizes limited amounts of formalin-fixed, paraffin-embedded tumor tissue to assess BRCAness status in both ER-positive and ER-negative breast cancer (BC) is currently lacking.

**Methods:**

A digital multiplex ligation-dependent probe amplification (digitalMLPA) assay was designed to detect copy number alterations required for the classification of BRCA1-like and BRCA2-like BC. The BRCA1-like classifier was trained on 71 tumors, enriched for triple-negative BC; the BRCA2-like classifier was trained on 55 tumors, enriched for luminal-type BC. A shrunken centroid-based classifier was developed and applied on an independent validation cohort. A total of 114 cases of a randomized controlled trial were analyzed, and the association of the classifier result with intensified platinum-based chemotherapy response was assessed.

**Results:**

The digitalMLPA BRCA1-like classifier correctly classified 91% of the BRCA1-like samples and 82% of the BRCA2-like samples. Patients with a BRCA-like tumor derived significant benefit of high-dose chemotherapy (adjusted hazard ratio (HR) 0.12, 95% CI 0.04–0.44) which was not observed in non-BRCA-like patients (HR 0.9, 95% CI 0.37–2.18) (*p* = 0.01). Analysis stratified for ER status showed borderline significance.

**Conclusions:**

The digitalMLPA is a reliable method to detect a BRCA1- and BRCA2-like pattern on clinical samples and predicts platinum-based chemotherapy benefit in both triple-negative and luminal-type BC.

## Background

Breast cancers arising in women with a *BRCA1* or *BRCA2* germline mutation are characterized by genomic instability. As both BRCA1 and BRCA2 play a role in the process of homologous recombination, non-functioning BRCA genes result in incorrect DNA repair, leading to gross genomic instability. Previously, our group has shown that *BRCA1*- and *BRCA2*-mutated tumors have a specific pattern of chromosomal gains and losses [1–3]. These specific genomic regions were used to develop a BRCA1-like and a BRCA2-like classifier. In follow-up studies, we showed that these classifiers could be used to identify germline *BRCA1*- or *BRCA2*-mutated cases, but also tumors with other mechanisms of *BRCA1* or *BRCA2* inactivation such as BRCA1 promoter methylation [1, 4, 5]. In addition, the classifiers have been used in the classification of *BRCA1* variants of unknown significance (VUS) [2].

Interestingly, we showed that the classifiers could not only be used to identify mechanisms of BRCA inactivation, but could also be used to predict treatment benefit. We hypothesized that a BRCA1-like or BRCA2-like profile is a read-out for a homologous recombination deficiency (HRD) phenotype and could therefore indicate tumors which would be highly sensitive to DNA-damaging agents. In several studies, we showed a remarkable benefit of high-dose alkylating chemotherapy in patients with a BRCA1-like or BRCA2-like profile [6–9]. In addition to high-dose chemotherapy, a gene expression-based BRCA1ness test, derived from the DNA-based BRCA1-like classifier, could predict benefit from the addition of veliparib/carboplatin to paclitaxel in the ISPY2 trial [10]. The currently presented BRCA-like test could function as a companion diagnostic test.

For a clinical test, it is essential that the method is highly reliable, has a fast turnaround time, and works on small quantities of paraffin-embedded tissue material. Multiplex ligation-dependent probe amplification (MLPA) is an established method and a standardized assay applied in routine diagnostics worldwide [11–15]. MLPA is a method based on the amplification and relative quantification of the ligated adjacent probes, which can target up to 50 different genomic regions that show diagnostically or clinically significant copy number changes in patient samples [11]. There are several advantages of MLPA over other copy number profiling methods: its fast turnaround time, suitable for degraded paraffin-embedded material, and use of standard (diagnostic) molecular laboratory equipment. In the past, we developed a multiplex ligation-dependent probe amplification (MLPA) assay to perform a BRCA1-like classification based on 34 MLPA probes [16]. The BRCA2-like copy number profile contains threefold as many genomic regions as the BRCA1-like profile, making it impossible to design a MLPA. Recently, digitalMLPA was introduced as a novel technology to measure copy number aberrations on 700 genomic locations [17], making it a perfect technique for combined BRCA1-like and BRCA2-like profiling.

In the current study, we developed a digitalMLPA assay to assess both the BRCA1-like and BRCA2-like patterns. We validated the digitalMLPA assay on an independent sample cohort. In addition, we profiled samples from a randomized controlled trial [18], to determine if the digitalMLPA has treatment predictive value. We showed that the assay could identify both triple-negative and luminal-type tumors with a remarkable good survival after high-dose double-strand break (DSB)-inducing chemotherapy.

## Methods

### Sample selection

Three series of breast cancer specimens were used for this study. (1) For the development and testing of the BRCA1-like classifier, a set of samples enriched for triple-negative (TN) breast cancer was used, as was done in the original BRCA1-like classifier development study [2]. (2) For the BRCA2-like classification, we used a mixed set of different subtypes, similar to the original BRCA2 classifier study [1]. For both the training and the test sets, samples were equally divided and stratification was based on ER status, material type (paraffin-embedded or fresh frozen), and array-based BRCA-like classification. Samples were scored as positive for ER and/or PR by immunohistochemistry (IHC), when at least 10% of the tumor cell nuclei showed staining of the ER or PR, respectively. A sample was scored as being HER2 positive when either a strong membrane staining (3+) could be observed by IHC or if CISH revealed amplification of HER2 in samples with moderate (2+) membrane staining at IHC. Mutation/methylation status was available for a minority of the patients that had undergone clinical genetic testing. The other patients are indicated as “unknown.” Supplemental figure 1 shows the sample flow through the training and validation series for the BRCA1-like and BRCA2-like digitalMLPA development. (3) To assess whether the digitalMLPA BRCA1-like and BRCA2-like classifiers had treatment predictive value, we analyzed samples from the Dutch high dose trial [18]. In this trial, patients were randomized between standard dose 5-fluorouracil-epirubicin-cyclophosphamide (FEC) chemotherapy and high-dose cyclophosphamide-thiotepa-carboplatin (HD-CTC) chemotherapy. We were able to include samples from 122 women for the digitalMLPA analysis; as for quite some trial patients, no tissue blocks or DNA was left for further analysis. Table 1 gives an overview of the sample series. The institutional review board of The Netherlands Cancer Institute approved the study. All trial participants provided written informed consent. The issue of non-trial participants was obtained under an opt-out regime in this study conform to Dutch regulations and the Code of Conduct of Federa-COREON (https://www.federa.org/codes-conduct).

**Table 1:** Patient and sample characteristics

*A. Series 1 for development of the BRCA1 classifier*				
	**Training set**		**Validation set**	
	***N***	**%**	***N***	**%**
**Material type**				
Fresh frozen	27	38	24	34
FFPE	44	62	46	66
**ER status**				
ER neg	48	68	48	69
ER pos	21	30	21	30
Unknown	2	3	1	1
**HER status**				
HER2 neg	24	34	21	30
HER2 pos	4	6	3	4
Unknown	43	61	56	66
**Array-based BRCA1-like classification**				
Non-BRCA1-like	30	42	28	40
BRCA1-like	41	58	42	60
**Mutation status**				
BRCA1mut	8	11	8	11
BRCA2mut	2	3	3	4
Unknown	61	86	59	84
**BRCA1 promoter methylation**				
Non-methylated	39	55	36	51
BRCA1-methylated	14	20	12	17
Unknown	18	25	22	31
*B. Series 2 for development of the BRCA2 classifier*				
	**Training set**		**Validation set**	
	***N***	**%**	***N***	**%**
**Material type**				
Fresh frozen	21	38	21	38
FFPE	34	62	35	63
**ER status**				
ER neg	13	24	16	29
ER pos	40	73	38	68
Unknown	2	4	2	4
**HER status**				
HER2 neg	20	36	20	36
HER2 pos	5	9	4	7
Unknown	30	55	32	57
**Array-based BRCA2-like classification**				
Non-BRCA2-like	30	55	28	50
BRCA2-like	25	46	28	50
**Mutation status**				
BRCA1mut	1	13	1	13
BRCA2mut	7	88	7	88
Unknown	47		48	
*C. Series 3 for assessing treatment prediction power*				
	**FEC**	**HD-CTC**	**Total**	***p*value**
	***n*(%)**	***n*(%)**	***n*(%)**	
**Age**				
< 40	17 (32%)	20 (29%)	37 (30%)	0.84
> 40	36 (68%)	49 (71%)	85 (70%)	
**ER status**				
Neg	21 (40%)	22 (32%)	43 (35%)	0.45
Pos	32 (60%)	47 (68%)	79 (65%)	
**PR status**				
Neg	30 (57%)	28 (41%)	58 (48%)	0.10
Pos	23 (43%)	41 (59%)	64 (52%)	
**pT-stage**				
1	6 (11%)	16 (23%)	22 (18%)	**0.03**
2	37 (70%)	49 (71%)	86 (70%)	
3	10 (19%)	4 (6%)	14 (11%)	
**Grade**				
I	6 (12%)	13 (21%)	19 (17%)	0.17
II	16 (31%)	25 (40%)	41 (35%)	
III	29 (57%)	25 (40%)	54 (47%)	
**No. of positive lymph nodes**				
< 10	34 (64%)	47 (68%)	81 (66%)	0.70
≥ 10	19 (36%)	22 (32%)	41 (34%)	
**Array-based BRCA1-like classification**				
Non-BRCA1-like	39 (74%)	56 (81%)	95 (78%)	0.38
BRCA1-like	14 (26%)	13 (19%)	27 (22%)	
**Array-based BRCA2-like classification**				
Non-BRCA2-like	40 (75%)	53 (77%)	93 (76%)	1.00
BRCA2-like	13 (23%)	16 (23%)	29 (24%)	
*D. Clinical variables in patients included in the digitalMLPA analysis and in the total HER2-negative trial population*				
	**digitalMLPA tested,*n*(%)**	**digitalMLPA not tested,*n*(%)**	***p*value**	
***n***	122	500		
**Age (median (SD))**	44.00 (6.96)	44.67 (6.12)	0.28	
**Treatment**				
CONV	53 (43.4)	249 (49.8)	0.25	
HD	69 (56.6)	251 (50.2)		
**ER status**				
Negative (< 10%)	43 (35.2)	106 (21.2)	**< 0.01**	
Positive (≥ 10%)	79 (64.8)	393 (78.6)		
NA	0 (0.0)	1 (0.2)		
**PR status**				
Negative (< 10%)	58 (47.5)	170 (34.0)	**0.01**	
Positive (≥ 10%)	64 (52.5)	319 (63.8)		
NA	0 (0.0)	11 (2.2)		
**pT-stage**				
1	22 (18.0)	129 (25.8)	0.05	
2	86 (70.5)	290 (58.0)		
3	14 (11.5)	79 (15.8)		
NA	0 (0.0)	2 (0.4)		
**Grade**				
Grade I	19 (15.6)	121 (24.2)	0.05	
Grade II	41 (33.6)	192 (38.4)		
Grade III	54 (44.3)	176 (35.2)		
NA	8 (6.6)	11 (2.2)		
**No. of positive lymph nodes**				
LN < 10	81 (66.4)	324 (64.8)	0.82	
LN ≥ 10	41 (33.6)	176 (35.2)		
**Array-based BRCA1-like classification**				
Non-BRCA1-like	95 (77.9)	115 (23.0)	0.05	
BRCA1-like	27 (22.1)	16 (3.2)		
NA	0 (0.0)	369 (73.8)		
**Array-based BRCA2-like classification**				
Non-BRCA2-like	93 (76.2)	105 (21.0)	0.27	
BRCA2-like	29 (23.8)	22 (4.4)		
NA	0 (0.0)	373 (74.6)		

*FFPE* formalin-fixed, parafin-embedded, *ER* estrogen-receptor, *PR* progesterone receptor, *pT-stage* pathological Tumor size, *FEC* 5-fluorouracil-epirubicin-cyclophosphamide, *HD-CTC* high-dose cyclophosphamide-thiotepa-carboplatin, *CONV* conventional dose 5-fluorouracil-epirubicin-cyclophosphamide, *HD* high-dose cyclophosphamide-thiotepa-carboplatin, *NA* not available

### Development of digitalMLPA probe mix for BRCAness

Array comparative genomic hybridization (Array-CGH)-based classifiers recognizing genomic alterations specific for BRCA1-mutated and BRCA2-mutated tumors were developed and previously published [1, 2]. Array CGH was performed on a microarray containing 3.5 bacterial artificial chromosomes (BACs). Using the shrunken centroids algorithm, BAC clones located in the most important regions contributing to the respective classifiers were identified for both a BRCA1-like and a BRCA2-like pattern. For each BAC clone selected based on high centroid value, at least one digitalMLPA probe was designed to be included in the BRCAness digitalMLPA assay. This resulted in a set of 84 digitalMLPA probes for BRCA1ness and 206 digitalMLPA probes for BRCA2ness classification (Supplemental Table 1).

In addition to the BRCAness-specific probes, a set of 206 “digital karyotyping” probes was included that covers all chromosome arms. This set was used for determining gross chromosomal aberrations and for data normalization. A set of 128 internal quality control probes was also included to determine reaction quality, amount of input DNA, and sample contamination and for troubleshooting purposes [17]. Quality tests were performed at MRC Holland to test the performance of each probe included in the BRCAness digitalMLPA assay (product details available at request). Probes showing high variability in DNA samples with normal copy numbers (standard deviation > 0.1) or probes sensitive to pipetting mistakes or technical errors during digitalMLPA experiments were replaced by other probes that performed better in the quality tests.

### digitalMLPA experiments

digitalMLPA experiments were performed as described previously [17]. Briefly, 40 ng of each DNA test sample was mixed with 2 μL of a unique barcode solution (MRC Holland), followed by DNA denaturation. Reactions were incubated with the digitalMLPA D004-X2 BRCAness probemix overnight at 60 °C to ensure hybridization of the probes to the sample DNA. The next day, the probes were ligated and amplified by PCR. The PCR-amplified products were then loaded onto an Illumina MiSeq sequencer (Illumina, San Diego, CA) for quantification using the MiSeq Reagent Kit version 3 (150 cycles; Illumina). Reference samples included were triplicates of Promega Human Genomic male DNA (Promega Benelux, Leiden, the Netherlands).

### Data analysis

Analysis of MiSeq data was done as described previously [17]. First, reads were mapped to the corresponding digitalMLPA probes present in the probemix. After correct identification, read counts for each probe were first compared with the median value of all reference probes in each sample. In the second step, the normalized values of each individual probe were compared with the median value of the corresponding probe in the reference samples. These final ratios were used as input for class prediction.

### Class prediction

Data values from all the 290 probes of interest (84 digitalMLPA probes for BRCA1-like and 206 digitalMLPA probes for BRCA2-like classification) were used for prediction analysis for microarrays (PAM) in R. In brief, the nearest shrunken centroid method [19] was applied to the training set with maximum distinct clinical courses (first set), and the classification performance was evaluated by a 10-times-repeated 10-fold cross-validation. A cutoff was chosen to make the best discrimination (highest accuracy) between a BRCA1-like and a non-BRCA1-like profile for the BRCA1-like classifier and between a BRCA2-like and a non-BRCA2-like profile for the BRCA2-like classifier (supplementary figure 2).

Subsequently, the obtained classifiers were tested on the respective test sets using the predefined cutoffs. The BRCA1 classifier and BRCA2-like results obtained by array CGH [1, 2] were considered as the gold standard.

### Statistics

In the Dutch high dose trial, we performed the Kaplan-Meier method and compared survival by log-rank tests (using R version 3.6 and package Survminer). Cox regression analysis was used to calculate hazard ratios in 114 trial patients where clinical information was complete; for 8 patients, histological grade was unknown. Multivariate Cox regression models were stratified for the number of lymph nodes and TN status and adjusted for pT-stage, histological grade, and BRCA-like status. The interaction with treatment was calculated between BRCA-like and non-BRCA-like patients adjusted as mentioned above. These analyses were repeated for the patients with a triple-negative tumor as well as for the ER/PR-positive Her2-negative (luminal-type) tumors.

This study was designed according to the REporting recommendations for tumor MARKer prognostic studies (REMARK) guidelines (Supl table 3) [20].

## Results

### Training and validation of digitalMLPA-based BRCAness classifiers

Table 1 gives an overview of the sample series used to develop (training set) and test (test set) the classifier. We tested how the digitalMLPA BRCA1-like classifier performed against the array-based test result, which was considered the gold standard. A threshold for the digitalMLPA BRCA1-like was set at 0.14 using the array-based test result (supplemental figure 2A). Using this threshold, the BRCA1-like classification based on the digitalMLPA data resulted in an accuracy of 99% in the training set (Table 2). All BRCA1-like were correctly classified, and only one non-BRCA1-like sample was scored as a BRCA1-like sample with digitalMLPA. Performance in the test set was excellent with an accuracy of 91%. Six samples were not correctly classified by digitalMLPA using the specified cutoff values: three BRCA1-like and three non-BRCA1-like samples. Two of the misclassified samples had a classifier result just below the threshold. Two other samples had a copy number pattern with many chromosomal aberrations and some noise, due to a lower quality DNA sample. For the other two misclassified samples, we could not find an explanation. The digitalMLPA assay performed equally well on paraffin-embedded or fresh frozen samples (data not shown).

**Table 2:** Results of the digitalMLPA BRCA1 classifier in the training and test sets

	***Predicted with digitalMLPA***		Class error rate (%)
	BRCA1-like	Non-BRCA1-like	
***Array-based score***			
**Training set**			
BRCA1-like	41	0	0
Non-BRCA1-like	1	29	3
Overall error rate			1
**Test set**			
BRCA1-like	39	3	7
Non-BRCA1-like	3	25	10
Overall error rate			9
**Performance in the test set**			
Sensitivity = 93%			
Specificity = 90%			
Accuracy = 91%			

### Results of the classifier in the prediction of BRCA2-like status

For BRCA2-like classification, the cutoff was set at 0.21, using the already established BRCA2-like array-based classification (Supplemental figure 2B). The BRCA2-like classifier had an accuracy of 89% in the training set; four BRCA2-like and two non-BRCA2-like samples were not correctly classified (Table 3). In the test set, the accuracy was 82%; seven BRCA2-like and three non-BRCA2-like samples were misclassified.

**Table 3:** Results of the digitalMLPA BRCA2 classifier in the training and test sets

	***Predicted with digitalMLPA***		Class error rate (%)
	BRCA2-like	Non-BRCA2-like	
***Array-based score***			
**Training set**			
BRCA2-like	21	4	16
Non-BRCA2-like	2	28	7
Overall error rate			11
**Test set**			
BRCA2-like	21	7	25
Non-BRCA2-like	3	25	11
Overall error rate			18
**Performance in test set**			
Sensitivity = 75%			
Specificity = 89%			
Accuracy = 82%			

### Classifier performance in BRCA-mutated and BRCA-methylated samples

As a next step, we assessed if the digitalMLPA classifiers correctly classified samples with a *BRCA1* mutation, *BRCA2* mutation, and *BRCA1* promoter methylation (Table 4). As previously shown by us, the BRCA1-like and BRCA2-like classifiers detected 88–90% of BRCA1-mutated, BRCA1-methylated, or BRCA2-mutated tumors [2, 4, 5]. The digitalMLPA classified 88% of the *BRCA1*-mutated tumors and 96% of BRCA1-methylated tumors as BRCA1-like. Ninety-three percent of the *BRCA2-*mutated tumors were classified as BRCA2-like.

**Table 4:** Classification of BRCA1-mutated, BRCA1 promoter methylation, and BRCA2-mutated samples

	*N* with correct classification
**BRCA1-like classification**	
*BRCA1*-mutated	88% (14/16)
*BRCA1* promoter methylation	96% (26/27)
**BRCA2-like classification**	
*BRCA2*-mutated	93% (13/14)

### Survival analysis Dutch high dose study

Most importantly, we tested if the digitalMLPA assay had treatment predictive value. In the Dutch high dose trial, patients were randomized between conventional FEC-based chemotherapy and HD-CTC. We previously showed that patients with BRCA1-like and BRCA2-like tumors had remarkably better responses on HD-CTC than on FEC [8, 21]. To test if the digitalMLPA BRCAness assay could be used for treatment response prediction, we analyzed 122 samples of the same trial using digitalMLPA (Table 1 (C, D)). Median follow-up for these 122 patients was 8 years. After 5 years, 88 out of 122 patients were alive (72%) and 76 out of 122 did not have a recurrence within 5 years (data not shown). Supplemental Table 2 shows the association of BRCA1- and BRCA2-like tumors with known clinical risk factors in trial patients. The digitalMLPA BRCA1-like pattern was associated with younger age, ER-negative tumors, and high grade, as previously shown by us using array-based BRCAness classification methods [5, 21, 22]. The BRCA2-like profile was associated with ER- and PR-positive tumors.

**Fig. 1:**
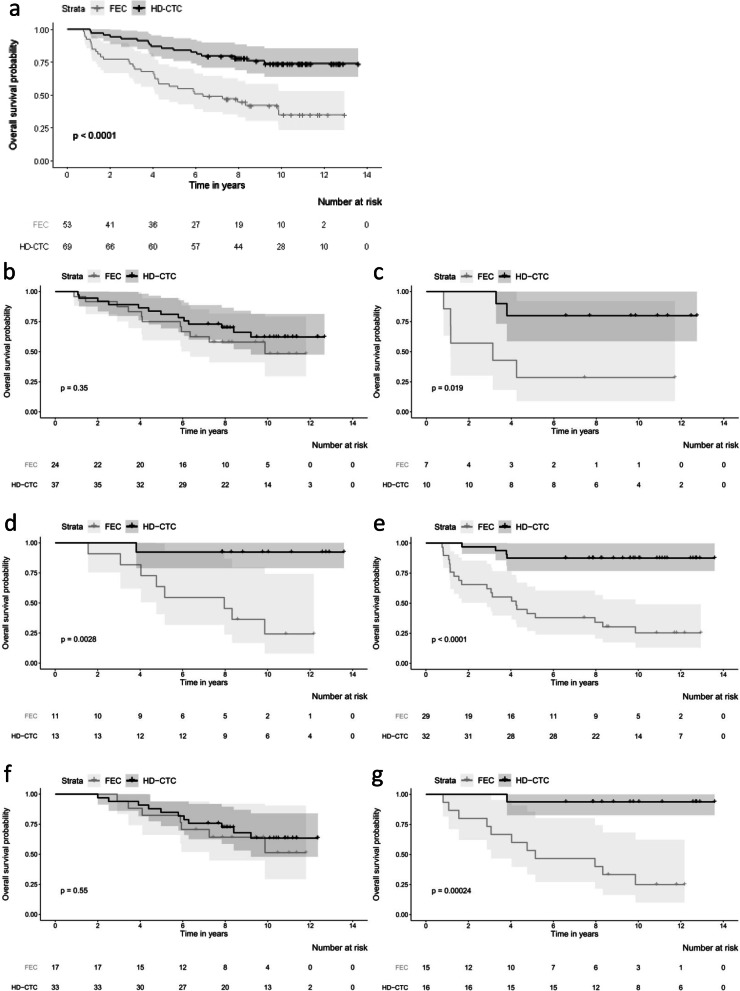
Kaplan-Meier survival plots for overall survival according to digitalMLPA BRCAness status and stratified for treatment in the Dutch high-dose study. Survival is subsequently shown all patients (**a**), for non-BRCA-like patients (**b**), BRCA1-like patients (**c**), BRCA2-like patients (**d**), BRCA-like patients (**e**), ER+ non-BRCA-like patients (**f**), and ER+ BRCA-like patients (**g**). *p* values have been obtained by log-rank test

Figure 1 shows Kaplan-Meier plots for overall survival for all patients, non-BRCA-like, BRCA1-like, BRCA2-like, and BRCA-like (either a BRCA1-like or a BRCA2-like profile) tumors, stratified for treatment. HR in this subset of the original trial population was 0.38 (95% CI 0.20–0.72; *p* = 0.003) for HD-CTC compared to FEC chemotherapy. Patients with a BRCA-like profile had a significantly better overall survival after HD-CTC treatment compared with FEC chemotherapy (adjusted HR 0.12, 95% CI 0.04–0.44, *p* = 0.001) (Table 5). This difference was not observed in non-BRCA-like patients (adjusted HR 0.90, 95% CI 0.37–2.18; *p* = 0.818). The effect of HD-CTC over FEC treatment was significantly different between BRCA-like and non-BRCA-like patients (*p* interaction 0.011). The different effect of treatment for BRCA-like and non-BRCA-like tumors was borderline significant when the analysis was stratified for triple-negative and luminal breast cancer (*p* interaction was 0.072 and 0.07, respectively) (Table 5 and Fig. 1). Subgroup analysis should be interpreted with caution, due to small numbers.

**Table 5:** Multivariate Cox proportional-hazard analysis of the risk of death and digitalMLPA BRCAness status in all patients, patients with TN tumors, and patients with HR-positive tumors

Variable	All 114 patients with 47 events				37 TN patients with 17 events				77 HR pos patients with 30 events			
	No. events/no. patients	Hazard ratio	95% CI	*p* value	No. events/no. patients	Hazard ratio	95% CI	*p* value	No. events/no. patients	Hazard ratio	95% CI	*p* value
**pT-stage**												
pT1/pT2	36/100	1.00			12/31	1.00			24/69	1.00		
pT3	11/14	2.87	1.35–6.08	0.006	5/6	1.92	0.63–5.87	0.249	6/8	1.71	1.50–10.94	0.06
**Histologic grade**												
I/II	21/60	1.00			3/8	1.00			18/52	1.00		
III	26/54	1.96	1.01–3.81	0.0478	14/29	1.97	0.52–7.47	0.318	12/25	1.25	0.96–4.78	0.064
**digitalMLPA**												
Non-BRCA-like tumor	23/58	1.00			5/10	1.00			18/48	1.00		
BRCA-like tumor	24/56	0.94	0.50–1.76	0.845	12/27	1.18	0.29–2.50	0.765	12/29	1.79	0.44–2.05	0.906
**BRCA-like tumor**												
FE_90_C chemotherapy	21/29	1.00			10/14	1.00			11/15	1.00		
HD-CTC chemotherapy	3/27	0.12	0.04–0.44	0.001	2/13	0.15	0.03–0.73	0.0185	1/14	0.09	0.01–0.80	0.0311
**Non-BRCA-like**		*p* interaction = 0.011*				*p* interaction = 0.072				*p* interaction = 0.070		
FE_90_C chemotherapy	10/22	1.00			3/6	1.00			7/16	1.00		
HD-CTC chemotherapy	13/36	0.90	0.37–2.18	0.818	2/4	0.91	0.10–8.02	0.934	11/32	0.75	0.28–1.97	0.558

Three separate multivariate Cox regression models were run in all patients (*n* = 114 patients with complete clinical variables), in patients with TN tumors, and in patients with HR-pos tumors^§^ (see top row) and an *interaction term with treatment. The first model was stratified for number of lymph nodes (4–9 vs. ≥ 10) and triple-negative status (ER < 10% and PR < 10% vs. others) and based on 114 patients. For patients with TN tumors (37 patients) and with HR-pos tumors (77 patients) only, models were stratified for lymph node status. *Test of homogeneity of treatment-specific hazard ratios based on an interaction term. *TN* triple-negative, *HR-pos* hormone receptor-positive, *pT-stage* pathological tumor size, *FE*_*90*_*C* 5-fluorouracil-epirubicin-cyclophosphamide, *HD-CTC* high-dose cyclophosphamide-thiotepa-carboplatin

## Discussion

In this study, we developed a novel digitalMLPA assay to assess BRCA1-like and BRCA2-like copy number profiles within one assay. We showed an accuracy of 91% for the BRCA1-like classification and 82% for the BRCA2-like classification using the array-based BRCA-like classification as the gold standard. The digitalMLPA correctly classified most *BRCA1*-mutated, *BRCA1*-methylated, and *BRCA2*-mutated tumors, with respectively 88%, 96%, and 93% correct classification. The digitalMLPA had treatment predictive power: the test could identify both TN and luminal-type tumors with remarkable good responses on high-dose chemotherapy.

The BRCA1-like and BRCA2-like classifiers were originally developed to identify *BRCA1* and *BRCA2* deficiency and could support decision-making in genetic counseling. We previously showed that the classifiers could identify tumors with other mechanisms of *BRCA1* or *BRCA2* inactivation such as promoter methylation [1, 4, 5]. In addition to the role of the test in clinical genetics decision-making, the BRCA1-like and BRCA2-like classifiers have shown to have treatment predictive value [6–8, 21]. As BRCA1 and BRCA2 play a role in the process of DNA double-strand break repair, we believe that a copy number profile resembling *BRCA1*- or *BRCA2*-mutated tumors could indicate the specific chromosomal instability associated with defective DNA repair, i.e., HRD. A BRCA1-like or BRCA2-like profile could therefore be considered as a readout for HRD. In addition to our BRCA1/2-like tests, other (commercially) available HRD tests are on the market for breast and ovarian cancer. These are either based on genomics scars [23], mutational signatures [24], or point mutations in homologous recombination repair genes [25]. In ovarian cancer, the highest benefit of niraparib was seen in patients with HRD tumors [26, 27]. In breast cancer, trials in the (neo) adjuvant setting showed higher responses on platinum salts or alkylating chemotherapy for patients with HRD tumors vs. patients with non-HRD tumors [28, 29]. However, the lack of a control arm was a flaw in these studies [30, 31]. Of note, a randomized trial in the neoadjuvant setting comparing paclitaxel single agent with cisplatin single agent showed higher responses for HRD tumors in both arms, indicating that this test is not predictive for selective benefit of DNA-damaging agents [32]. Furthermore, in the advanced breast cancer TNT trial, no association was observed between HRD status and response rate [33]. In this latter trial, an additional explanation for genomic scars having less predictive power is that most patients were already pre-treated by other chemotherapy agents. Resistance might already have been occurred in these patients [34]. Although patients with BRCAness tumors might have benefit from conventional types of DNA-damaging therapy, like FEC, we believe that BRCAness patients will have additional benefit of high-dose DNA-damaging therapies, like the regimen applied in the Dutch high dose trial, as these are in particular highly vulnerable for this kind of tumors [6, 7]. Results of in vitro and in vivo studies point in the same direction [35].

Our BRCA1-like and BRCA2-like classifiers are breast cancer subtype specific. The BRCA1-like profile is mainly observed in TN tumors, while the BRCA2-like profile is mainly observed in luminal-type cancer. As TN and luminal-type breast tumors have distinct molecular profiles, developing two distinct classifiers, based on each subtype-specific genomic profiles, resulted in highly specific classifiers. The BRCA1-like classifier has been validated in three different studies as a predictor of high-dose chemotherapy benefit [6–8]. Currently, a phase III trial is running in stage 3 triple-negative breast cancer, applying the BRCA1-like MLPA test (NCT02810743). For luminal-type breast cancer, the digitalMLPA is an easy-to-use, robust assay, to assess the BRCA2-like phenotype. With such a test, we could easily test retrospective series and generate data on lumina-type breast cancer, which is currently lacking in the literature due to the focus on TN tumors. There is evidence from the literature that a small group of luminal tumors may also have benefit from HRD-inducing chemotherapy. Manie et al. showed that HRD testing using genomic instability assays in luminal-type tumors resulted in a threefold increase in the identification of HRD tumors than by HRD testing solely based on *BRCA* mutation profiling [36]. Our BRCA2-like test has the potential to identify a subpopulation of luminal-type tumors that might benefit from high-dose DSB-inducing chemotherapy. The fact that the BRCA1-like and BRCA2-like tests are combined in one assay facilitates routine testing in a diagnostic lab.

Advantages of digitalMLPA, as compared to array- or NGS-based BRCA-like testing, include a high dynamic range for copy number detection, a robust assay, the good performance on degraded DNA, and the low input amount (≥ 20 ng) of DNA [17]. Especially, in patient samples with limited tissue material, such as core biopsies, a low input amount is a prerequisite. With digitalMLPA, a large number of genomic loci of interest can be analyzed for copy number alterations in a single reaction with low hands-on time, and results are available within 36 h. Because of the targeted approach, data analysis and result interpretation are easier than with array-based analysis and can be performed in any routine diagnostic setting.

A limitation of the current study is that we could not profile the whole randomized trial population, due to missing tissue blocks or a limited amount of DNA available. The series profiled was enriched for triple-negative and BRCA1-like tumors, as these were the samples with enough DNA left for successful digital MLPA analysis (Table 1 (D)). As we know that BRCA1-like tumors have a very low HR (ranging from 0.05 to 0.19 [6, 7]) for high-dose chemotherapy, the overall HR (0.38) in our study population is lower than in the total HER2-negative trial population (HR 0.68) [37]. However, even in this selected subgroup, we showed a significant interaction between treatment benefit and digitalMLPA BRCAness status, indicating the strong predictive power of our digitalMLPA assay. Independent validation of a different cohort will be necessary; we are currently exploring possibilities. Another suggestion for improvement would be to train a specific classifier to predict treatment benefit. However, the BRCA-like classifiers are based on the biology of BRCA tumors and thus are not merely “data-driven,” which could be seen as a strength. The third limitation is the slightly worse performance of the BRCA2-like classifier. We noticed before that BRCA2-like tumors are more alike sporadic luminal-type tumors. It appeared more difficult to find specific discriminatory regions [1, 38].

## Conclusions

DigitalMLPA BRCAness analysis shows results comparable to previously published array-based BRCA-like testing. The digitalMLPA is a fast and reliable method to detect copy number changes in relevant regions for BRCA1- and BRCA2-like classification in one experiment in breast cancer samples. We showed that the digitalMLPA assay had treatment predictive power, in both TN and luminal-type tumors. Independent validation of patient cohorts treated with DNA DSB-inducing chemotherapy is warranted.

## Supplementary information

**Additional file 1: Figure 1.** Consort diagram for training and validation the BRCA1 –like (A) and BRCA2-like (B) digitalMLPA classifiers. The Number of correctly classified samples by digitalMLPA is indicated as well as the number of BRCA1 mutated, BRCA1 methylated and BRCA2 mutated samples. **Figure 2.** Determination of cut-off for BRCA1-like (A) and BRCA2-like (B) classification in training set. Cut-off value for BRCA1-like was set 0.14 and cut-off value for BRCA1-like was set at 0.21. **Table 2.** Association of BRCA-like test result and clinical characteristics.

**Additional file 2:** Proble list digitalMLPA BRCAness probemix.

**Additional file 3:.** Description of the study according to REMARK criteria.

## Data Availability

The dataset supporting the conclusions of this article is available upon reasonable request. Requests should be made to Dr. E.H. Lips (e.lips@nki.nl).

## Abbreviations

BC: Breast cancer
BAC: Bacterial artificial chromosome
CGH: Comparative genomic hybridization
FEC: 5-Fluorouracil-epirubicin-cyclophosphamide
HD-CTC: High-dose cylclophosphamide-thiotepa-carboplatin
HR: Hazard ratio
HRD: Homologous recombination deficiency
MLPA: Multiplex ligation-dependent probe amplification
TN: Triple-negative
VUS: Variants of unknown significance
