# Supplementary material for: BRCAness digitalMLPA profiling predicts benefit of intensified platinum-based chemotherapy in triple-negative and luminal-type breast cancer
Source: Breast Cancer Res. 2020 Jul 25;22:79. doi: 10.1186/s13058-020-01313-7 (PMC7382055; doi:10.1186/s13058-020-01313-7)
Supplement: Supplementary file 1 — Additional file 1: Figure 1. Consort diagram for training and validation the BRCA1 –like (A) and BRCA2-like (B) digitalMLPA classifiers. The Number of correctly classified samples by digitalMLPA is indicated as well as the number of BRCA1 mutated, BRCA1 methylated and BRCA2 mutated samples. Figure 2. Determination of cut-off for BRCA1-like (A) and BRCA2-like (B) classification in training set. Cut-off value for BRCA1-like was set 0.14 and cut-off value for BRCA1-like was set at 0.21. Table 2. Association of BRCA-like test result and clinical characteristics. [file 13058_2020_1313_MOESM1_ESM.docx]

**Supplements**

Supplemental figure 1

A.

B.

Supplemental figure 1. Consort diagram for training and validation the BRCA1 –like (A) and BRCA2-like (B) digitalMLPA classifiers. The Number of correctly classified samples by digitalMLPA is indicated as well as the number of BRCA1 mutated, BRCA1 methylated and BRCA2 mutated samples.

Supplemental figure 2

A.

B.


*Supplemental figure 2.* Determination of cut-off for BRCA1-like (A) and BRCA2-like (B) classification in training set. Cut-off value for BRCA1-like was set 0.14 and cut-off value for BRCA1-like was set at 0.21.

*Supplemental table 2.* Association of BRCA-like test result and clinical characteristics.

|  |  | non BRCA-like | BRCA1-like | BRCA2-like | BRCA1-like and BRCA2-like | p-value |
| --- | --- | --- | --- | --- | --- | --- |
|  |  | n(%) | n(%) | n(%) | n(%) |  |
| Age | <40 | 16 (26%) | 11 (65%) | 4 (17%) | 6 (30%) | **0.01** |
|  | >40 | 45 (75%) | 6 (35%) | 20 (83%) | 14 (70%) |  |
| ER | Neg | 13 (21%) | 15 (88%) | 1 (4%) | 14 (70%) | **<0.01** |
|  | Pos | 48 (79%) | 2 (12%) | 23 (96%) | 6 (30%) |  |
| PR | Neg | 21 (34%) | 16 (94%) | 5 (21%) | 16 (80%) | **<0.01** |
|  | Pos | 40 (66%) | 1 (6%) | 19 (79%) | 4 (20%) |  |
| pTstage | 1 | 9 (16%) | 4 (29%) | 4 (17%) | 5 (25%) | 0.8 |
|  | 2 | 46 (84%) | 10 (71%) | 17 (71%) | 13 (65%) |  |
|  | 3 | 6 (9%) | 3 (17%) | 3 (13%) | 2 (10%) |  |
| BRgrade | I | 14 (22%) | 0 | 5 (22%) | 0 | **<0.01** |
|  | II | 25 (39%) | 2 (11%) | 9 (35%) | 5 (28%) |  |
|  | III | 19 (30%) | 13 (72%) | 9 (35%) | 13 (72%) |  |
|  | Unknown | 3 | 3 | 1 | 2 |  |
| LN | <10 | 55 (90%) | 14 (82%) | 18 (75%) | 17 (85%) | 0.4 |
|  | ≥10 | 6 (10%) | 3 (18%) | 6 (25%) | 3 (15%) |  |
