# Supplementary material for: BRCAness digitalMLPA profiling predicts benefit of intensified platinum-based chemotherapy in triple-negative and luminal-type breast cancer
Source: Breast Cancer Res. 2020 Jul 25;22:79. doi: 10.1186/s13058-020-01313-7 (PMC7382055; doi:10.1186/s13058-020-01313-7)
Supplement: Supplementary file 3 — Additional file 3:. Description of the study according to REMARK criteria. [file 13058_2020_1313_MOESM3_ESM.docx]

**Supplemental Table 3**

Description of the study according to REMARK criteria.

|  |  |
| --- | --- |
| REporting recommendations for tumour MARKer prognostic studies (REMARK) |  |
|  |  |
| **Introduction** |  |
| 1. State the marker examined, the study objectives, and any prespecified hypotheses. | The marker examined is BRCA1-like and BRCA2-like status determined by digitalMLPA. The study objective was to develop and validate a digitalMLPA based BRCA1-like and BRCA2-like test. We hypothesized that using a digitalMLPA test with 290 specific chromosomal markers this would be feasible. |
| **Materials and Methods** |  |
| **Patients** |  |
| 2. Describe the characteristics (e.g. disease stage or comorbidities) of the study patients, including their source and inclusion and exclusion criteria. | Patients fromhospital based cohorts were used to develop and validate the BRCA-like and BRCA2-like classifiers. All samples had been profiled before with arrayCGH. The samples were a mix of the different breast cancer subtypes (ER+, TN) and both FFPE-derived and fresh frozen samples were included. For treatment effect prediction samples were used from the Dutch high dose chemotherapy trial (Rodenhuis, NEJM 2003). |
| 3. Describe treatments received and how chosen (e.g. randomised or rule-based). | The patients used for classifier development and validation were treated by surgery. Patients that were treated in the randomized controlled trial were treated with either high dose chemotherapy containing 4 cycles of FEC induction followed by one cycle of cyclophosphamide, thiotepa and carboplatin or as conventional regimen 5 cycles of FEC. |
|  |  |
| **Specimen characteristics** |  |
| 4. Describe type of biological material used (including control samples), and methods of preservation and storage. | DNA isolated from FFPE archival tissue or fresh frozen tissue was used for digitalMLPA analysis. |
|  |  |
| **Assay methods** |  |
| 5. Specify the assay method used and provide (or reference) a detailed protocol, including specific reagents or kits used, quality control procedures, reproducibility assessments, quantitation methods, and scoring and reporting protocols. Specify whether and how assays were performed blinded to the study end point. | The protocol has been described in Benard-Slagter et al (J Mol Diag 2017, reference no. 17). Cut-offs for BRCA1-like and BRCA2-like classification were empirically chosen to make the best discriminiation between BRCA-like and non-BRCA-like. The arraCGH test result was considered as the gold standard. |
|  |  |
| **Study design** |  |
| 6. State the method of case selection, including whether prospective or retrospective and whether stratification or matching (e.g. by stage of disease or age) was employed. Specify the time period from which cases were taken, the end of the follow-up period, and the median follow-up time. | See methods section of this paper. Samples for the BRCA1-like test development were enriched for TN status. Samples for BRCA2-like test development were enriched for luminal-type breast cancer. Samples were equally split between training and validation series. All patients characteristics are shown in table 1. For the randomized controlled trial patients selection was based upon availability of DNA. Median follow up for the trial patients was 8 years. |
| 7. Precisely define all clinical end points examined. | Overall Survival was used as the endpoint to study the assays capability to predict treatment benefit. |
| 8. List all candidate variables initially examined or considered for inclusion in models. | Standard prognostic variables: ER, T stage, number of tumor positive lymph nodes, Bloom Richardson grade |
| 9. Give rationale for sample size; if the study was designed to detect a specified effect size, give the target power and effect size. | For the classifier development we used large sample series. Samples were equally split between training and validation sets. For the randomized trial cohort we analysed the largest number possible. We do note that some of the survival analyses for the subtypes have a low power, due to low sample numbers. |
|  |  |
| **Statistical analysis methods** |  |
| 10. Specify all statistical methods, including details of any variable selection procedures and other model-building issues, how model assumptions were verified, and how missing data were handled. | Fisher exact test was performed to compare clinical variables between groups. For BRCA1-like and BRCA2-like classifier development a nearest neighbor shrunken centroids based method (Prediction analysis of rmicroarrays (PAM)) was performed. For survival analysis the Kaplan Meier method was applied and cox multivariate regression models were built. |
| 11. Clarify how marker values were handled in the analyses; if relevant, describe methods used for cutpoint determination. | BRCA1-like and BRCA2-like based on array-CGH were considered as gold standard, and the digitalMLPA BRCA1-like and BRCA2-like classifiers were developed against these arrayCGH classifiers. Cut-offs were based on maximal discrimination between arrayCGH BRCA-like and arrayCGH non-BRCA-like samples. Consequently, the digitalMLPA BRCA1-like classifier had a cut-off of 0.14 and the digitalMLPA BRCA2-like classifier had a cut-off of 0.21. |
|  |  |
| **Results** |  |
| **Data** |  |
| 12. Describe the flow of patients through the study, including the number of patients included in each stage of the analysis (a diagram may be helpful) and reasons for dropout. Specifically, both overall and for each subgroup extensively examined report the numbers of patients and the number of events. | Supplemental figure 1 represents a consort diagram, and shows the flow of the samples through the study. |
| 13. Report distributions of basic demographic characteristics (at least age and sex), standard (disease-specific) prognostic variables, and tumour marker, including numbers of missing values. | Table 1 shows the basic clinical variables of the three cohorts used. |
|  |  |
| **Analysis and presentation** |  |
| 14. Show the relation of the marker to standard prognostic variables. | Supplemental table 1 shows the association between the digitalMLPA BRCA1-like and BRCA2-like scores and standard prognostic variables. |
| 15. Present univariate analyses showing the relation between the marker and outcome, with the estimated effect (e.g. hazard ratio and survival probability). Preferably provide similar analyses for all other variables being analysed. For the effect of a tumour marker on a time-to-event outcome, a Kaplan–Meier plot is recommended. | Tables 2, 3 and 4 show the digitalMLPA scores for the training and validation sets, and for BRCA1 mutated, BRCA1 methylated and BRCA2 mutated breast cancer. Figure 1 shows Kaplan-Meier analysis for the randomized trial samples. Supplemental table 1 shows the association of the BRCA-like tests with standard clinical variables. |
| 16. For key multivariable analyses, report estimated effects (e.g. hazard ratio) with confidence intervals for the marker and, at least for the final model, all other variables in the model. | Table 5 shows multivariate cox proportional hazard analysis of the risk of death and digitalMLPA BRCAness status. |
| 17. Among reported results, provide estimated effects with confidence intervals from an analysis in which the marker and standard prognostic variables are included, regardless of their significance. | Table 5 shows multivariate models, with correction for standard prognostic variables. |
| 18. If done, report results of further investigations, such as checking assumptions, sensitivity analyses, internal validation. | We ran our multivariate cox models separately for TN and Luminal-type (HR-positive) tumors. |
|  |  |
| **Discussion** |  |
| 19. Interpret the results in the context of the prespecified hypotheses and other relevant studies; include a discussion of limitations of the study. | In the discussion section of the manuscript we describe that we were successful in developing and validating digitalMLPA classifiers with an accuracy of 91% and 82% for BRCA1-like and BRCA2-like classification, respectively. In addition, we showed that our assay had treatment predictive value in both TN as well as luminal-type breast cancer, although subgroup analysis was based on small numbers. We discuss limitations of the study, and place our results in the context of other studies in this field. |
| 20. Discuss implications for future research and clinical value. | In the discussion section, we discuss the clinical relevance of the study, and discuss a possible application in routine diagnostics. We discuss that based on our study, independent validation in large clinical cohorts with treatment data available is warranted. |
